# Pathological roles of bone marrow adipocyte-derived monocyte chemotactic protein-1 in type 2 diabetic mice

**DOI:** 10.1038/s41420-023-01708-3

**Published:** 2023-11-13

**Authors:** Shan Wan, Jinwei Xie, Yan Liang, Xijie Yu

**Affiliations:** 1 Laboratory of Endocrinology and Metabolism/Department of Endocrinology and Metabolism, Rare Disease Center, West China Hospital, Sichuan University, Chengdu, China; 2 General Practice Ward/International Medical Center Ward, General Practice Medical Center, West China Hospital, Sichuan University, Chengdu, China; 3 Department of Orthopedic Surgery, West China Hospital, Sichuan University, Chengdu, China; 4 Core Facilities of West China Hospital, Sichuan University, Chengdu, China

**Keywords:** Chemokines, Islets of Langerhans

## Abstract

Type 2 diabetes mellitus (T2DM) has become a prevalent public health concern, with beta-cell dysfunction involved in its pathogenesis. Bone marrow adipose tissue (BMAT) increases in both the quantity and area in individuals with T2DM along with heightened monocyte chemotactic protein-1 (MCP-1) secretion. This study aims to investigate the influence and underlying mechanisms of MCP-1 originating from bone marrow adipocytes (BMAs) on systemic glucose homeostasis in T2DM. Initially, a substantial decrease in the proliferation and glucose-stimulated insulin secretion (GSIS) of islet cells was observed. Moreover, a comparative analysis between the control (Ctrl) group and *db/db* mice revealed significant alterations in the gene expression profiles of whole bone marrow cells, with a noteworthy upregulation of *Mcp-1*. And the primary enriched pathways included chemokine signaling pathway and AGE-RAGE signaling pathway in diabetic complications. In addition, the level of MCP-1 was distinctly elevated in BMA-derived conditional media (CM), leading to a substantial inhibition of proliferation, GSIS and the protein level of phosphorylated Akt (p-Akt) in Min6 cells. After blocking MCP-1 pathway, we observed a restoration of p-Akt and the proliferation of islet cells, resulting in a marked improvement in disordered glucose homeostasis. In summary, there is an accumulation of BMAs in T2DM, which secrete excessive MCP-1, exacerbating the abnormal accumulation of BMAs in the bone marrow cavity through paracrine signaling. The upregulated MCP-1, in turn, worsens glucose metabolism disorder by inhibiting the proliferation and insulin secretion of islet cells through an endocrine pathway. Inhibiting MCP-1 signaling can partially restore the proliferation and insulin secretion of islet cells, ultimately ameliorating glucose metabolism disorder. It’s worth noting that to delve deeper into the impact of MCP-1 derived from BMAs on islet cells and its potential mechanisms, it is imperative to develop genetically engineered mice with conditional *Mcp-1* knockout from BMAs.

## Introduction

T2DM is a collective term encompassing a range of endocrine and metabolic disorders primarily characterized by abnormal glucose metabolism due to a relative insufficiency of insulin. The international diabetes federation (IDF) diabetes atlas 10th edition reports a continued global increase in diabetes prevalence, affecting 537 million people worldwide [https://diabetesatlas.org/]. Existing research indicates the involvement of various internal and external factors in the onset and progression of T2DM [1, 2]. The principal contributor of T2DM is insulin resistance triggered by obesity, affecting liver, adipose, and skeletal muscle. As a consequence, islet beta-cells gradually lose their compensation capacity, leading to inadequate insulin secretion [3]. Hence, investigations aimed at enhancing the functionality of islet beta-cells are of paramount importance in the treatment of diabetes and its associated complications.

Bone marrow adipose tissue (BMAT) constitutes a physiological component of bone marrow and comprises ~70% of the total bone marrow volume in healthy adults [4, 5]. In the past, scientists primarily considered BMAT as a relatively inert filling tissue within the bone marrow cavity [5, 6]. However, more recent research has unveiled the existence of two distinct forms of BMAT: constitutive bone marrow adipose tissue (cMAT) and regulated bone marrow adipose tissue (rMAT), both of which play integral roles in local and systemic metabolic regulation, effectively functioning as an endocrine organ [6, 7]. In individuals with T2DM, BMAT experiences a significant increase in both its quantity and area [8, 9].

A previous study has provided evidence of substantial alterations in the gene expression profile of bone marrow mesenchymal stem cells (BMSCs) in individuals with T2DM. Specifically, genes associated with adipogenesis, such as CCAAT/enhancer-binding protein α (*Cebpα*), peroxisome proliferator-activated receptor γ (*Pparγ*), adiponectin (*Adipoq*), and fatty acid-binding protein 4 (*Fabp4*), exhibited significant upregulation. Conversely, anti-adipogenesis genes, such as sirtuin 1 (*Sirt1*) and GLI family zinc finger 1(*Gli1*), were notably downregulated [9]. Furthermore, in T2DM, the transcriptome and secretion profile of BMA also underwent substantial changes [9]. For instance, the adipokines leptin (*LEP*) and resistin (*RETN*) were markedly upregulated, while insulin-like growth factor 1 (*IGF1*) and *ADIPOQ* exhibited significant downregulation. In addition, the cytokines *MCP-1* and matrix metalloproteinase 2 (*MMP2*) were distinctly upregulated, with *MCP-1* being one of the primary factors affected. Importantly, this phenomenon appears to be highly conserved [9].

Factors released by hypertrophic BMA in individuals with T2DM have the potential to induce nearby BMSCs to undergo adipogenesis *via* paracrine signaling [9], ultimately promoting energy storage. It’s worth noting that individuals with T2DM often experience activation of hyperglycemia and oxidative stress within the bone marrow microenvironment [8, 10]. These conditions may serve as initiating factors for the dysregulated adipogenesis and osteogenesis of BMSCs [11, 12]. Research has also shown that hyperglycemia and hypoxia can stimulate the expression of *Mcp-1* in adipocytes [12, 13]. MCP-1, a monomeric polypeptide, is synthesized by various cell types, including adipocytes, endothelial cells, monocytes, smooth muscle cells, etc. [14, 15]. Typically, its production is triggered by inflammatory stimuli like interleukin-1 (IL-1), interleukin-4 (IL-4), or tumor necrosis factor-α (TNF-α) [14]. MCP-1 assumes a pivotal role in conditions such as insulin resistance, diabetes and related complications like diabetic nephropathy and retinopathy. Immune and inflammatory mechanisms hold significant significance within adipose tissue in cases of obesity [16], likely exerting an impact on the development of insulin resistance. MCP-1 plays a crucial role in recruiting monocytes and activating macrophages, potentially contributing to the initiation and perpetuation of inflammatory processes within adipose tissue [17]. In vitro investigations of adipocytes have revealed that oxidative stress induced by hyperglycemia can stimulate adipocytes to secrete LEP, MCP-1, and interleukin-6 (IL-6) [18]. Nevertheless, research concerning MCP-1 production by BMA and its implications in the development of bone marrow obesity and insulin resistance remains scarce. Consequently, this study aims to investigate the pathological role and potential mechanism of MCP-1 in T2DM, both in vivo and in vitro, with the aim of establishing a theoretical basis for the clinical prevention and treatment of T2DM.

## Results

### Accumulated adipocytes in bone marrow cavity and a markedly upregulated expression of *Mcp-1* in diabetic mice

To investigate aberrant gene expression in bone marrow cells of diabetic mice and its impact on glucose metabolism, we successfully generated a T2DM mouse model by employing a combination of high fat-diet (HFD) feeding and multiple low-dose streptozotocin (MLD-STZ) induction [19, 20]. In addition, we took meticulous care in our selection process by opting for spontaneously diabetic *db/db* mice to ensure that our experimental outcomes remained unaffected by any STZ-induced damage to rodent islet cells, as illustrated in Supplementary Fig. 1.

**Fig. 1:**
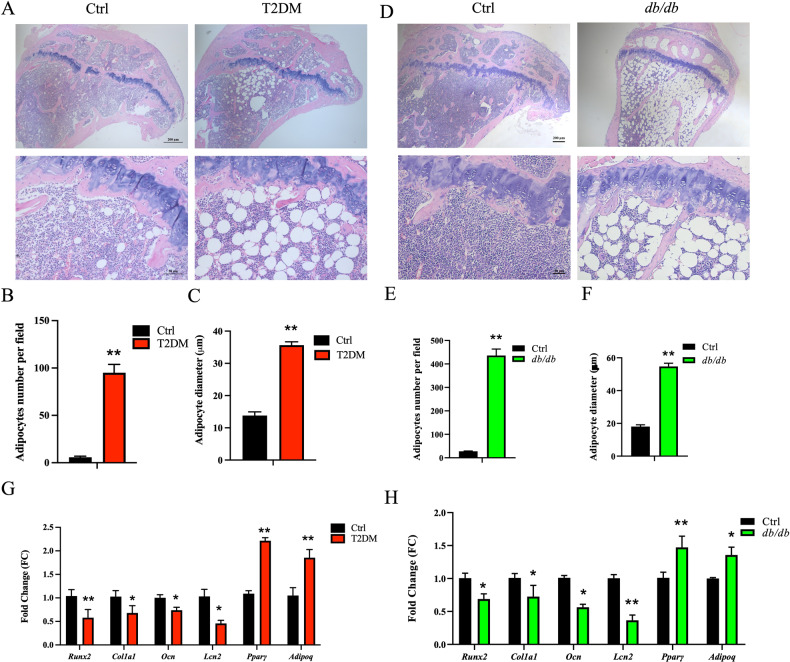
Marked accumulation of bone marrow adipose tissue in type 2 diabetic mice. **A**, **D** HE staining revealed a significant increase in BMAT in the proximal tibia of the T2DM and *db/db* groups (scale bar = 200, 50 μm from top to bottom). **B**, **C**, **E**, **F** Both the number and diameter of BMAs in the proximal tibia of T2DM and *db/db* mice were dramatically higher than those of the Ctrl group. **G**, **H** The mRNA expression levels of metaphyseal osteogenesis-related genes, including *Runx2*, *Col1a1*, *Ocn*, and *Lcn2*, were distinctly downregulated in the T2DM and *db/db* groups compared to the Ctrl group. In contrast, the adipogenesis-related genes *Pparγ* and *Adipoq* were memorably upregulated. *n* = 6. *p < 0.05, **p < 0.01.

Conventionally, BMAT has been believed to merely act as a passive filler in the bone marrow cavity [5, 6]. However, owing to its unique anatomical location, BMAT serves several distinct functions. In our study, we observed a significant 1530.0% increase in the number of BMAs in T2DM mice compared to the Ctrl group, accompanied by a substantial increase in BMA diameter (Fig. 1A–C). Similarly, in spontaneously diabetic *db/db* mice, both the number and diameter of BMAs exhibited markedly increases of 1482.9% and 203.4%, respectively, in comparison to the Ctrl group (Fig. 1D–F). These findings suggest that disturbances in glucose metabolism in T2DM can result in an augmentation of both the number and diameter of BMAs.

To elucidate the pathological mechanism behind the accumulation of BMAT in T2DM, we assessed the messenger RNA (mRNA) expression levels of genes related to osteogenesis and adipogenesis in the metaphyseal bone tissue. Our results revealed that, in comparison to the Ctrl group, the T2DM group exhibited significant downregulation of osteogenesis-related genes, namely, runt-related transcription factor 2 (*Runx2*), collagen type I alpha 1 (*Col1a1*), osteocalcin (*Ocn*) and lipocalin-2 (*Lcn2*), in the metaphyseal region. Conversely, the expression levels of adipogenesis-related genes, *Pparγ* and *Adipoq*, were notably upregulated (Fig. 1G). Similar findings were observed when we analyzed the same mRNA expression in the metaphyseal bone of *db/db* mice (Fig. 1H), suggesting that glucose metabolism disturbances inhibit osteogenesis while promoting adipogenesis in BMSCs.

**Fig. 2:**
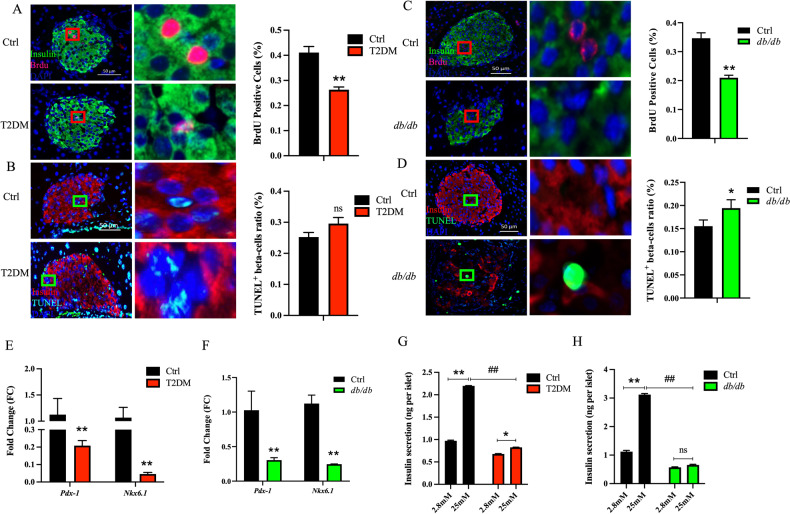
Signally attenuated proliferation and insulin secretion of islet beta-cells in type 2 diabetic mice. **A**, **C** The proliferation of islet beta-cells in T2DM and *db/db* groups attenuated compared to the Ctrl group (*n* = 3). **B**, **D** The apoptosis of islet beta-cells in T2DM and *db/db* groups increased (*n* = 6 and 3). **E**, **F** The mRNA expression levels of *Pdx-1* and *Nkx6.1* were significantly downregulated in islet cells from the T2DM and *db/db* groups compared to the Ctrl group (*n* = 8). **G**, **H** Basal insulin secretion and the GSIS of islet cells decreased overtly in T2DM and *db/db* group mice compared to the Ctrl group (*n* = 5 and 3). *p < 0.05 for intra-group comparison, **p < 0.01 for intra-group comparison, ##*p* < 0.01 for inter-group comparison, ns means no statistical difference.

To further illustrate the pathological mechanisms underlying metabolic disorders in diabetic mice, as illustrated in Supplementary Fig. 1, we employed Brdu/insulin and TUNEL/insulin double staining to assess the proliferation and apoptosis of islet cells, respectively. Our results demonstrated that the T2DM group showed a 38.5% reduction in islet cell proliferation compared to the Ctrl group (Fig. 2A), accompanied by a 17% increase in apoptosis (Fig. 2B). Consistent with these findings, beta-cells in the *db/db* group displayed a 39.5% reduction in proliferation (Fig. 2C) and a 25.1% increase in apoptosis (Fig. 2D). Furthermore, we evaluated the mRNA expression levels of key transcription factors, pancreatic and duodenal homeobox factor-1 (*Pdx-1*) and NK6 homeobox 1 (*Nkx6.1*), which are crucial determinants of pancreatic beta-cell proliferation. Our results indicated a significant downregulation of *Pdx-1* and *Nkx6.1* expression levels in the T2DM group compared to the Ctrl group (Fig. 2E). Similarly, the mRNA expression levels of *Pdx-1* and *Nkx6.1* in the *db/db* group were significantly lower than those in the Ctrl mice (Fig. 2F).

We proceeded to isolate mouse islet cells and collect supernatants from both basal (2.8 mM) and high glucose (25 mM) Krebs-Ringer bicarbonate HEPES buffer (KRBH buffer)-stimulated islet cells to further explore the underlying reasons for the glucose disorder in type 2 diabetic mice. Compared to the Ctrl mice, the basal insulin secretion of the T2DM mice decreased by 30.2%. Moreover, compared to their respective basal insulin secretion levels, the GSIS of the Ctrl mice increased by 126.1% (*p* < 0.01), while the GSIS of the T2DM mice increased by 21.7% (*p* < 0.05), signifying that the GSIS of the T2DM mice dramatically declined by 62.5% compared to the Ctrl mice (Fig. 2G). Similarly, basal insulin secretion in *db/db* group mice decreased by 49.4%, and the GSIS decreased by 79.3% (*p* < 0.01) when compared to the Ctrl group (Fig. 2H), indicating a substantial impairment in the insulin secretion function of mouse islet cells stimulated by high glucose in T2DM.

**Fig. 3:**
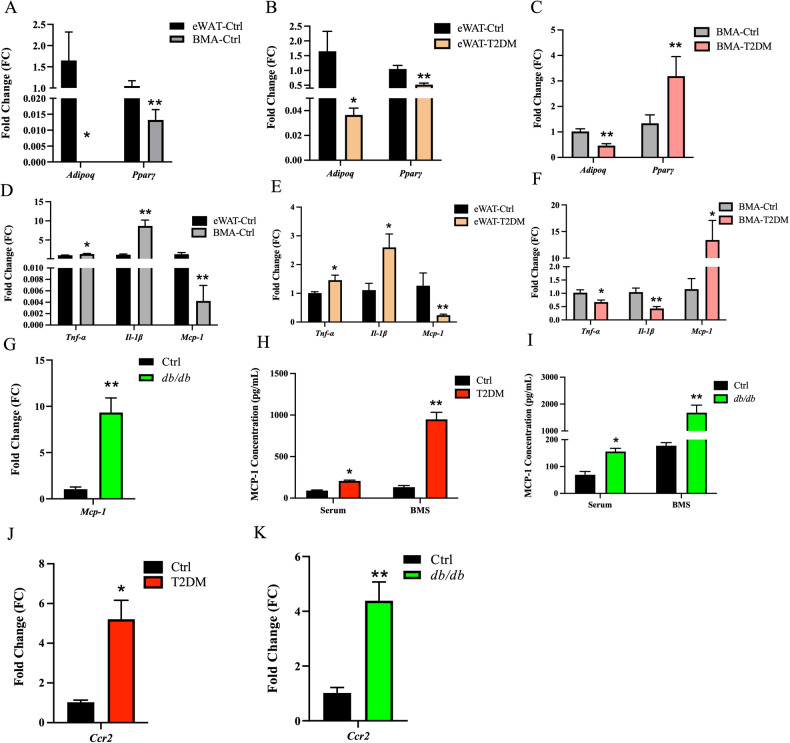
Dramatically upregulated mRNA level of *Mcp-1* in the bone marrow adipocytes of type 2 diabetic mice. **A** The expression levels of adipocyte-specific genes, *Adipoq* and *Pparγ*, in BMAs from the Ctrl mice were significantly lower than those in peripheral eWAT. **B**, **C** Compared to Ctrl mice, the expression levels of *Adipoq* and *Pparγ* in eWAT of T2DM mice were dramatically downregulated. The expression of *Adipoq* in BMAs was downregulated, while the level of *Pparγ* was distinctly increased. **D** The expression levels of inflammatory factors, *Tnf-α* and *Il-1β*, in BMAs of the Ctrl group were signally higher than those in peripheral eWAT, whereas the level of *Mcp-1* was overtly lower. **E**–**G** The expression levels of *Tnf-α* and *Il-1β* in the peripheral eWAT in the T2DM group were memorably upregulated compared to the Ctrl group, while the expression of *Mcp-1* was distinctly downregulated. The expression levels of *Tnf-α* and *Il-1β* in the BMAs of the diabetic group were significantly downregulated, whereas the expression of *Mcp-1* was observably upregulated. **H**, **I** The MCP-1 level in the serum and bone marrow supernatant of T2DM and *db/db* mice were significantly increased. **J**, **K** Compared to the Ctrl group, the expression of the MCP-1 receptor *Ccr2* in the pancreatic islet cells of the T2DM and *db/db* group was signally upregulated. *n* = 8. *p < 0.05, **p < 0.01.

Furthermore, we isolated BMA to evaluate the changes of its transcriptome. Previous study has established that BMA expresses adipose-specific genes, such as *Adipoq* and *Fabp4* [21]. In order to investigate the similarities and differences between BMA and white adipose tissue in T2DM, we examined the mRNA expression in epididymal white adipose tissue (eWAT) and BMA. Our results indicated that the expression levels of adipocyte-specific genes, *Adipoq* and *Pparγ*, in BMA were distinctly lower than those in eWAT in Ctrl mice (Fig. 3A). In addition, when compared to the Ctrl group, the expression levels of *Adipoq* and *Pparγ* in the peripheral eWAT of the T2DM group were signally downregulated by 97.8% and 50.5%, respectively (Fig. 3B). The mRNA level of *Adipoq* in BMA of T2DM mice decreased by 54.4%, while the expression of *Pparγ* increased markedly by 138.0% (Fig. 3C). These results indicate that the adipogenic capacity of BMA was substantially lower than that of peripheral eWAT in Ctrl mice but was memorably upregulated in BMA compared to eWAT in T2DM, contributing to the dramatic accumulation of BMAT in type 2 diabetic mice.

Next, we conducted a comparison of mRNA expression levels of inflammatory factors in eWAT and BMA. In the Ctrl group, the levels of *Tnf-α* and interleukin-1β (*Il-1β*) were notably higher in BMA than in peripheral eWAT, while the mRNA level of *Mcp-1* was much lower in BMA than that in eWAT (Fig. 3D). In T2DM mice, the mRNA expression levels of *Tnf-α* and *Il-1β* in eWAT were overtly upregulated, whereas the expression of *Mcp-1* was obviously downregulated compared to the Ctrl group (Fig. 3E). However, in contrast, compared to the Ctrl group, the mRNA expression levels of *Tnf-α* and *Il-1β* in the BMA of the T2DM group were downregulated, while the expression of *Mcp-1* was significantly upregulated (Fig. 3F). Similarly, we observed a distinct increase in the expression level of *Mcp-1* in the BMA of the *db/db* mice compared to the Ctrl group (Fig. 3G). These results suggest that T2DM mice exhibited a local inflammatory phenotype in peripheral eWAT, and elevated inflammatory factors inhibited differentiation of peripheral eWAT, ultimately leading to the development of insulin resistance in adipose tissue. However, the BMA of T2DM mice did not display an obvious inflammatory phenotype compared to peripheral eWAT; instead, *Mcp-1* was selectively upregulated in BMA.

In both diabetic patients and mouse models, serum MCP-1 levels have been shown to be significantly elevated [9]. In our present study, we observed a dramatical upregulation in the mRNA expression level of *Mcp-1* in BMA in T2DM (Fig. 3 and Supplementary Fig. 2). To further investigate this phenomenon, we measured the levels of MCP-1 in mouse serum and bone marrow supernatant (BMS). As depicted in Fig. 3H, compared to the Ctrl group, the MCP-1 levels in the serum and BMS of the T2DM group increased distinctly. Similarly, MCP-1 levels in serum and BMS of *db/db* mice were also increased when compared to the Ctrl group (Fig. 3I).

To explore the potential relationship between MCP-1 secretion by BMA and the changes in islet cells, we initially examined the mRNA expression level of the *Ccr2* receptor of MCP-1 in mouse islet cells. Our findings revealed a dramatical upregulation of *Ccr2* mRNA expression in the islet cells of the T2DM group compared to the Ctrl group (Fig. 3J, K), suggesting that the elevated MCP-1 may affect the islet cells in type 2 diabetic mice.

### Elevated *Mcp-1* mRNA expression in bone marrow cells from *db/db* mice

To investigate the alterations in gene expression profiles of bone marrow cells in T2DM and their pathological implications in the disease progression, transcriptome sequencing was performed on whole bone marrow cells collected from spontaneously diabetic *db/db* mice.

The transcriptome sequencing results revealed that, in comparison to the Ctrl mice, there were 2915 differentially expressed genes (DEGs) identified in the bone marrow cells of *db/db* mice, of which 1025 were upregulated and 1890 were downregulated (Supplementary Fig. 2A). Subsequently, we conducted a Kyoto Encyclopedia of Genes and Genomes (KEGG) analysis on these DEGs, which indicated that the enriched pathways included the phosphatidylinositol 3 kinase/protein kinase B (PI3K/Akt) signaling pathway, advanced glycation end product-receptor of advanced glycation end product (AGE-RAGE) signaling pathway in diabetic complications, chemokine signaling pathway and type II diabetes mellitus, *etc* (Supplementary Fig. 2B).

Upon analyzing the KEGG pathways mentioned above, we discovered that *Mcp-1* was involved in two diabetes-related pathways: the AGE-RAGE signaling pathway in diabetic complications and the chemokine signaling pathway (Supplementary Fig. 2C). We hypothesize that persistent hyperglycemia results in the non-enzymatic glycosylation of various proteins in the body, leading to the accumulation of AGEs. These AGEs subsequently bind to their receptors, RAGE, downregulate PI3K/Akt levels and ultimately upregulate *Mcp-1* mRNA expression mediated by nuclear factor kappa-B (NF-κB). These processes may play a crucial role in the development of chronic diabetes complications, including thrombosis, inflammation, and atherosclerosis (Supplementary Fig. 2D).

Moreover, within the chemokine signaling pathway, we observed an upregulation of the chemokine *Mcp-1*, which binds to its corresponding receptor chemokine (C-C motif) receptor 2 (CCR2), leading to the downregulation of PI3K/Akt through intracellular pathways. This, in turn, inhibits the phosphorylation of fork head box O (FOXO) and regulates cell proliferation, differentiation, and apoptosis (Supplementary Fig. 2E).

### Suppression of Min6 cell proliferation and insulin secretion function by MCP-1

**Fig. 4:**
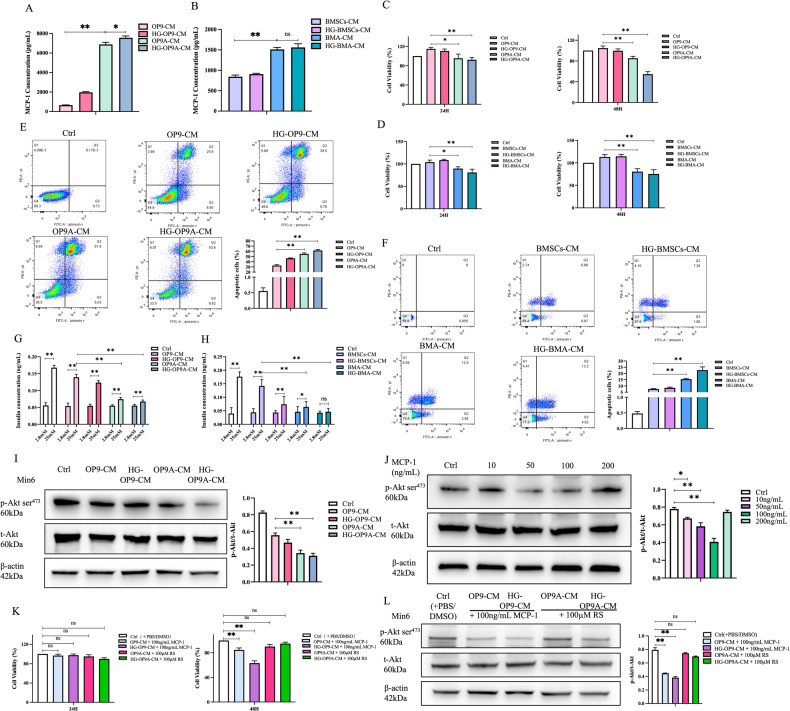
Impaired proliferation and the glucose-stimulated insulin secretion of Min6 cells through MCP-1/Akt pathway. **A**, **B** The MCP-1 levels in OP9A-CM/BMA-CM were overtly higher than those in OP9-CM/BMSCs-CM, with further increases observed in HG-OP9A-CM/HG-BMA-CM compared to OP9A-CM/BMA-CM (*n* = 16 and 12). **C**, **D** Compared to OP9-CM/BMSCs-CM, OP9A-CM/BMA-CM significantly inhibited the proliferation of Min6 cells at 24 h and 48 h of co-culture, and HG-OP9A-CM/HG-BMA-CM further inhibited Min6 cells proliferation (*n* = 12). **E**, **F** Compared to OP9-CM/BMSCs-CM, OP9A-CM/BMA-CM significantly aggravated the apoptosis of Min6 cells, and HG-OP9A-CM/HG-BMA-CM exacerbated the apoptosis of Min6 cells (*n* = 6). **G** GSIS in Min6 cells increased by 2-fold under Ctrl condition, 1.6-fold with OP9-CM intervention, and 1.3, 0.4 and 0.2-fold with HG-OP9-CM, OP9A-CM and HG-OP9A-CM interventions, respectively (*n* = 12). **H** GSIS in Min6 cells increased by 2.2-fold after BMSCs-CM intervention, while HG-BMSCs-CM, BMA-CM and HG-BMA-CM interventions resulted in increases of 0.7, 0.4 and 0.09-fold, respectively (*n* = 8). **I** The expression levels of t-Akt in Min6 cells in different intervention groups did not change dramatically compared to each other. Whereas the protein levels of p-Akt in Min6 cells were obviously decreased in the OP9A-CM/HG-OP9A-CM intervention group compared to the OP9-CM intervention group (*n* = 5). **J** Treatment with 10, 50 and 100 ng/mL of MCP-1 resulted in a gradual decrease in p-Akt protein levels of in Min6 cells compared to the Ctrl group (*n* = 5). **K** Compared to the Ctrl + PBS group, OP9-CM/HG-OP9-CM + 100 ng/mL Mcp-1 significantly decreased the proliferation of Min6 cells. Conversely, pretreatment of Min6 cells with the MCP-1 receptor CCR2 antagonist RS (100 µM), followed by co-culture with OP9A-CM/HG-OP9A-CM for 24 or 48 h, did not significantly alter cell viability (*n* = 12). **L** The protein level of p-Akt in Min6 cells after OP9-CM/HG-OP9-CM + 100 ng/mL MCP-1 intervention was obviously decreased compared to the Ctrl group, while the p-Akt protein level of Min6 cells regained to a level comparable to that of the Ctrl group when RS was added to block MCP-1 pathway (*n* = 5). *p < 0.05, **p < 0.01, ns means no statistical difference.

Previous study has revealed that BMA can affect glucose metabolism both directly and indirectly [6]. To explore the direct relationship between BMA and pancreatic islet cells, we employed in vitro cell models. Initially, we assessed the level of MCP-1 in various CM. As displayed in Fig. 4A, this analysis demonstrated that MCP-1 concentrations were low in OP9-CM and high glucose (HG)-OP9-CM. Conversely, the level of MCP-1 in OP9-derived adipocyte (OP9A)-CM was significantly higher than in OP9-CM, and the level of MCP-1 in HG-OP9A-CM was further increased compared to OP9A-CM. Similarly, the MCP-1 levels in primary BMSCs-CM and HG-BMSCs-CM were low. Whereas the level of MCP-1 in BMA-CM was dramatically higher than that in BMSCs-CM, and MCP-1 level in HG-BMA-CM was further elevated compared to BMA-CM (Fig. 4B). These results further confirmed the high expression of MCP-1 in BMA, with its expression further amplified under HG stimulation. Subsequently, we utilized HG-OP9A-CM/HG-BMA-CM to investigate their effects on the proliferation, apoptosis and GSIS of the mouse insulinoma cell line-Min6 cells.

After 24 and 48 h of co-culture, it was observed that compared to Min6 cells cultured with OP9-CM, OP9A-CM caused a notable inhibition in the proliferation of Min6 cells by 17.0% and 18.6%. Moreover, HG-OP9A-CM further restrained the proliferation of Min6 cells by 19.6 and 47.9% (Fig. 4C). Similarly, BMA-CM was found to be more effective than BMSCs-CM in inhibiting the proliferation of Min6 cells. Notably, HG-BMA-CM was observed to repress the proliferation of Min6 cells (Fig. 4D) further significantly, indicating that both BMA-derived CM are effective in inhibiting the proliferation of Min6 cells, with the inhibitory effect being further aggravated under HG stimulation.

Compared to OP9-CM, OP9A-CM was observed to increase the apoptosis of Min6 cells. In addition, HG-OP9A-CM further exacerbated the apoptosis of Min6 cells (Fig. 4E). Likewise, BMA-CM increased the apoptosis of Min6 cells in comparison to BMSCs-CM. HG-BMA-CM further intensified apoptosis in the Min6 cell line (Fig. 4F). These results indicate that both BMA-derived CM promoted the apoptosis of Min6 cells, with HG stimulation further promoting this effect.

Regarding the impact of CM on GSIS, it was observed that Min6 cells in the Ctrl group exhibited a 2-fold increase in GSIS. The OP9-CM intervention group showed a 1.6-fold increase, while the HG-OP9-CM, OP9A-CM and HG-OP9A-CM intervention groups exhibited increases of 1.3-, 0.4-, and 0.2-fold, respectively (Fig. 4G). Notably, OP9A-CM treatment resulted in a significant 46.6% decrease in GSIS compared to OP9-CM, while HG-OP9A-CM caused a marked 51.6% decrease. Similarly, BMA-CM intervention led to a significant 54.5% decrease in GSIS compared to BMSCs-CM, and HG-BMA-CM resulted in a clear 67.2% decrease (Fig. 4H). These results hint that both BMA-derived CM significantly impede the insulin secretion capacity of Min6 cells, with their inhibitory effects being further potentiated under HG conditions.

### Inhibition of Min6 cell proliferation through the MCP-1/PI3K/Akt pathway

Through transcriptome sequencing, we found a significant upregulation in the mRNA expression level of *Mcp-1* in bone marrow cells from *db/db* mice compared to the Ctrl group (Supplementary Fig. 2). The upregulated MCP-1 is believed to regulate cell proliferation via the MCP-1/CCR2/PI3K/Akt signaling (Supplementary Fig. 2E). To further investigate this, we conducted western blot (WB) analysis to detect Akt protein levels in Min6 cells after intervention with different CM. The results showed no significant difference in the protein levels of total Akt (t-Akt) in Min6 cells among the various CM intervention groups. However, in comparison to the OP9-CM group, the protein level of phosphorylated Akt (p-Akt) in Min6 cells was significantly downregulated after OP9A-CM intervention, and this downregulation was further pronounced in the HG-OP9A-CM intervention group (Fig. 4I).

Next, we examined the protein levels of p-Akt and t-Akt in Min6 cells after exogenous MCP-1 intervention. The results showed a downregulation in the protein levels of p-Akt in Min6 cells with 10, 50 and 100 ng/mL Mcp-1 intervention compared to the Ctrl group (Fig. 4J). Interestingly, the intervention with 200 ng/mL MCP-1 recombinant protein only slightly reduced the protein level of p-Akt in Min6 cells compared to the Ctrl group but significantly increased by 83.1% compared to the 100 ng/mL group, suggesting that the effect of MCP-1 on the p-Akt level in Min6 cells is concentration-dependent in vitro.

Our results demonstrate that both BMA-derived CM and exogenous MCP-1 can directly reduce the phosphorylation level of Akt in Min6 cells. To further investigate this issue, we simulated or blocked the MCP-1 pathway. In the co-culture system between OP9-CM/HG-OP9-CM or OP9A-CM/HG-OP9A-CM and Min6 cells, we intervened by adding MCP-1 recombinant protein or its receptor antagonist RS504393 (RS), respectively. The results showed that adding 100 ng/mL MCP-1 recombinant protein to the co-culture system between OP9-CM/HG-OP9-CM and Min6 cells had no significant effect on cell proliferation after 24 h, but decreased cell proliferation after 48 h compared to the Ctrl + PBS group (Fig. 4K). In contrast, blocking MCP-1 signaling with RS restored the proliferation ability of Min6 cells. WB showed that the protein levels of p-Akt in Min6 cells after OP9-CM/HG-OP9-CM + 100 ng/mL MCP-1 intervention were distinctly decreased compared to the Ctrl group (Fig. 4L). The above results suggest that BMA may inhibit the proliferation of Min6 cells by producing MCP-1 to downregulate Akt signaling.

### Blocking the MCP-1/PI3K/Akt signaling to ameliorate glucose metabolism disorders in diabetic mice

**Fig. 5:**
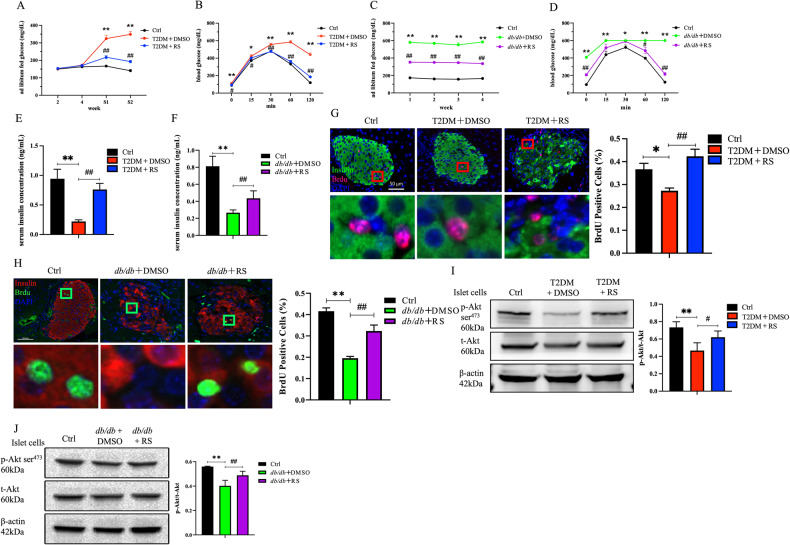
Blockade of MCP-1/Akt pathway improved glucose metabolism disorders and the p-Akt level in diabetic mice. **A** The ad libitum fed glucose levels of the T2DM + DMSO group were significantly elevated in S1 and S2 after STZ intervention compared to the Ctrl group. However, the ad libitum fed glucose levels in the T2DM + RS group showed a marked decrease at S1 and S2 compared to the T2DM + DMSO group. **B** The blood glucose levels at each time point during IPGTT in the T2DM + DMSO group were clearly higher than those in the Ctrl mice, however, the blood glucose levels in the T2DM + RS group were obviously declined compared to the T2DM + DMSO group. **C** Ad libitum fed glucose levels were dramatically elevated in *db/db* + DMSO mice compared to the Ctrl group, while they were signally reduced in *db/db* + RS mice compared to the *db/db* + DMSO group. **D** The changes in blood glucose at each time point during IPGTT after different treatments in *db/db* mice were consistent with those in the T2DM mice (*n* = 6). **E**, **F** The serum insulin levels in the T2DM + DMSO/*db/db* + DMSO groups were distinctly reduced compared to the Ctrl group, while the serum insulin levels in the T2DM + RS/*db/db* + RS group were increased compared to the T2DM + DMSO/*db/db* + DMSO group (*n* = 10 and 8). **G**, **H** Compared to the Ctrl mice, the proliferation of islet cells in the T2DM + DMSO/*db/db* + DMSO mice was markedly reduced, and the administration of the MCP-1 receptor antagonist to block the MCP-1 signaling in vivo overtly increased the proliferation of islet cells in the T2DM + RS/*db/db* + RS mice (*n* = 5). **I**, **J** Compared to the Ctrl group, the level of p-Akt in the pancreatic islet cells of the T2DM + DMSO/*db/db* + DMSO group was significantly downregulated. However, the level of p-Akt in the pancreatic islet cells of the T2DM + RS/*db/db* + RS group was partially restored and significantly upregulated compared to the T2DM + DMSO/*db/db* + DMSO group (*n* = 5). *p < 0.05 for intra-group comparison, **p < 0.01 for intra-group comparison, #*p* < 0.05 for inter-group comparison, ##*p* < 0.01 for inter-group comparison, ns indicates no statistical difference.

Compared to the Ctrl group, the ab libitum fed glucose levels of the T2DM + DMSO mice were significantly increased at first week (S1) and second week (S2). However, the T2DM + RS group showed a dramatic 32.9% and 44.7% decrease in ab libitum fed glucose levels at S1 and S2, compared to T2DM + DMSO mice (Fig. 5A). In addition, the blood glucose levels of T2DM + DMSO mice were significantly higher than those of the Ctrl mice at each time point of intraperitoneal glucose tolerance trial (IPGTT), whereas the blood glucose levels of the T2DM + RS group were signally lower than those of the T2DM + DMSO group, with a reduction of 57.8% at 120 minutes (Fig. 5B). Similarly, ab libitum fed glucose were markedly elevated in spontaneously diabetic *db/db* + DMSO mice compared to Ctrl mice but were obviously low in *db/db* + RS mice (Fig. 5C). During IPGTT, the blood glucose levels of *db/db* + DMSO group mice were distinctly higher than those of Ctrl mice at each time point, with a significant increase of 388.3% at 120 min. In contrast, the blood glucose levels of *db/db* + RS mice were distinctly lower than those of *db/db* + DMSO mice at 0, 60 and 120 min, with a reduction of 63.9% at 120 min (Fig. 5D).

Furthermore, the serum insulin level of the T2DM + DMSO group was significantly decreased compared to the Ctrl group, whereas the T2DM + RS group showed a marked increase compared to the T2DM + DMSO group (Fig. 5E). Similarly, the serum insulin level of the *db/db* + DMSO group was dramatically decreased compared to the Ctrl group, while the serum insulin level of the *db/db* + RS group was obviously elevated compared to the *db/db* + DMSO group (Fig. 5F).

After immunofluorescence staining of the mice pancreas, it was observed that the proliferation of islet cells in the T2DM + DMSO group was reduced by 25.6% compared to the Ctrl mice. Moreover, the T2DM + RS group showed an overt increase of 55.2% in islet cell proliferation compared to the T2DM + DMSO group (Fig. 5G). Blockade of MCP-1 signaling in *db/db* mice yielded similar results to those observed in T2DM mice (Fig. 5H).

In comparison to the Ctrl group, the T2DM + DMSO group exhibited a 36.4% reduction in p-Akt protein levels in the primary islet cells, while the T2DM + RS group showed an overt increase compared to the T2DM + DMSO group (Fig. 5I). Similarly, the blockade of the MCP-1 pathway in *db/db* mice resulted in similar outcomes to those observed in T2DM mice (Fig. 5J). Our results indicate that inhibiting MCP-1 signaling in type 2 diabetic mice can partially restore proliferation and insulin secretion of the islet beta-cells.

## Discussion and conclusion

### Accumulation of bone marrow adipose tissue in type 2 diabetes mellitus

The BMAT in the proximal tibia was visibly increased in both the HFD feeding plus MLD-STZ-induced T2DM and spontaneously homozygous mutant *db/db* diabetic mouse models. Hematoxylin-eosin (HE) staining revealed a significant enlargement in the diameter of BMA (Fig. 1A–F), indicating an enhanced potential for lipid storage and energy buffering [22]. Previous studies have reported the crucial roles of two adipocyte-specific genes, *Adipoq* [23] and *Pparγ* [24, 25], in bone metabolism. *Adipoq*, for instance, inhibits the proliferation and promotes the apoptosis of osteoblasts, ultimately leading to a reduction in bone mass and OCN levels. In contrast, *Pparγ* promotes the adipogenesis of BMSCs.

Interestingly, in the metaphyseal bone tissue of diabetic mice, the mRNA levels of *Adipoq* and *Pparγ* were upregulated, which in turn promoted the adipogenesis of BMSCs and the accumulation of adipocytes within the bone marrow cavity. Conversely, the expression levels of *Adipoq* and *Pparγ* in BMAT were significantly lower than those in peripheral eWAT under physiological conditions. However, in the T2DM group, the expression levels of *Adipoq* and *Pparγ* in the peripheral eWAT of mice were dramatically downregulated compared to the Ctrl group. In addition, there was a marked upregulation of inflammatory factors, including *Tnf-α* and *Il-1β*, in eWAT, leading to insulin resistance through various mechanisms [26–28]. Despite the downregulation of *Adipoq* in diabetic mice, the expression level of *Pparγ* within BMAT was significantly upregulated, thereby promoting the adipogenesis of BMSCs. This plays a crucial role in the increase of BMAT in the mouse model of T2DM, which subsequently negatively regulates osteogenesis, resulting in bone loss. Notably, hyperglycemia and oxidative stress serve as the initial triggers for the adipogenesis of BMSCs [8, 10], with reactive oxygen species accelerating the expansion of mitotic clones, thus to promoting the adipogenesis of BMSCs [11, 12].

Physiologically, adipose tissue, as the target organ of insulin, participates in glucose homeostasis within the body. Previous studies have reported a reduction in insulin sensitivity in peripheral eWAT, accompanied by an increase in insulin sensitivity observed in BMAT, particularly in obesity mouse models induced by a HFD feeding [29]. In our study, we observed a downregulation of the adipocytokine-*Pparγ* expression and a significant upregulation of inflammatory factors-*Tnf-α* and *Il-1β* within eWAT of T2DM mice. These changes contribute to insulin resistance by inhibiting adipocyte differentiation [26–28]. However, within BMAT, we observed a substantial upregulation in the expression of *Pparγ*, while the mRNA expression levels of *Tnf-α* and *Il-1β* were overtly downregulated. These findings demonstrate that BMAT does not exhibit an inflammatory phenotype in type 2 diabetic mice, which is in line with previous research reported by Tencerova et al. [29].

### Elevated *Mcp-1* expression in Bone marrow adipocyte of individuals with type 2 diabetes mellitus

Previous research has indicated that the expression of Mcp-1 is induced in adipocytes and other cell types in response to hyperglycemia and hypoxia, mediated by the reactive oxygen species system [12, 13, 17]. In our present study, we observed a distinct upregulation of *Mcp-1* in bone marrow cells from *db/db* mice. Subsequent KEGG enrichment analysis provided insights into the mechanisms behind this upregulation. It appears that persistent hyperglycemia leads to the non-enzymatic glycosylation of various proteins in the body, resulting in the accumulation of AGEs. These AGEs subsequently bind to their receptor, RAGE, which triggers downstream signaling events leading to the downregulation of PI3K/Akt through a series of intracellular reactions. This downregulation ultimately promotes the mRNA expression of *Mcp-1*, a process mediated by NF-κB. This upregulated MCP-1 may promote the occurrence and development of chronic complications associated with diabetes. In addition, when MCP-1 binds to its receptor CCR2, it is likely to regulate cell proliferation, differentiation, and apoptosis through the PI3K/Akt signaling.

In our subsequent experiments, we observed a significant increase in the mRNA expression of *Mcp-1* in BMA from both T2DM and *db/db* mice (Fig. 3F, G). Furthermore, the levels of MCP-1 were dramatically elevated in BMS of both T2DM and *db/db* mice (Fig. 3H, I). In addition, we noted an increase in MCP-1 levels in the supernatant of the OP9 cell line and primary BMSCs following adipogenic differentiation in vitro. Notably, this increase was further enhanced by exposure to HG conditions (Fig. 4A, B). These findings suggest a potential association between the expansion of BMA and the upregulation of MCP-1, with this effect being amplified under diabetic conditions.

Our study revealed that BMAT increases in T2DM, leading to excessive secretion of MCP-1. This surplus MCP-1, in turn, promotes adipogenesis and inhibits osteogenesis of BMSCs through paracrine signaling, thereby establishing a detrimental “*vicious cycle*” within the bone marrow microenvironment, ultimately leading to the accumulation of BMAT. Enrichment analysis further suggested that elevated MCP-1 levels may contribute to the occurrence and progression of chronic complications of diabetes, such as thrombosis, inflammation, and atherosclerosis. In addition, MCP-1 binds to chemokine receptors, leading to the downregulation of PI3K/Akt signaling, ultimately inhibiting cell proliferation and secretion (Supplementary Fig. 2D and E).

### The MCP-1/PI3K/Akt axis regulates the function of islet cells

In both T2DM and *db/db* mice, we observed an upregulation of the MCP-1 receptor CCR2 in pancreatic islet cells (Fig. 3J, K), alongside a significant downregulation of key transcription factors, *Pdx-1* and *Nkx6.1*, resulting in reduced islet cell proliferation of impaired GSIS (Fig. 2). These findings suggest a potential association between MCP-1 produced by BMA and the impaired function of islet cells. High levels of MCP-1 in OP9A-CM/BMA-CM and HG-OP9A-CM/HG-BMA-CM were found to dramatically inhibit the proliferation, accelerate the apoptosis, and impair GSIS of Min6 cells (Fig. 4C–H). The addition of the MCP-1 receptor antagonist RS helped correct the abnormal changes in the proliferative capacity of Min6 cells. Moreover, the addition of exogenous MCP-1 to the co-culture system led to a distinct attenuation of proliferation in Min6 cell (Fig. 4K). This study also revealed that MCP-1 secreted by BMA inhibited the protein level of p-Akt in Min6 cells, while blocking the Mcp-1 signaling in Min6 cells restored the level of p-Akt (Fig. 4I, L). These positive/negative co-culture experiments indicate that BMA can secrete MCP-1 to regulate the proliferation, apoptosis and GSIS of Min6 cells.

MCP-1 is a pivotal cytokine involved in immune and inflammatory responses. Animal studies have demonstrated that blocking the MCP-1/CCR2 axis can effectively reverse the progression of damage in organs such as lung, heart, and kidney [30–32]. In the tumor microenvironment, macrophages secrete MCP-1 to activate the PI3K/Akt signaling, promoting tumor endocrine resistance by recruiting monocytes into the tumor microenvironment [33]. PI3K/Akt is a critical regulator of cell survival during stress, governing cell proliferation and differentiation under both physiological and pathological conditions [34]. Dysregulation of this pathway can lead to severe disruptions in cell function. Akt kinase belongs to the kinase A, G, C (AGC kinase) family, and its downstream targets are involved in protein synthesis, glycogen metabolism and cell cycle modulation [35]. Akt, also known as protein kinase B (PKB), initiates a network that positively regulates G1/S cell cycle progression by inactivating glycogen synthase kinase 3β (GSK-3β), resulting in increased cyclin D1 levels and inhibition of FOXO [36]. This, in turn, plays a pivotal role in cell proliferation, differentiation, and apoptosis. Building upon our findings, we propose that MCP-1 secreted by BMA may inhibit proliferation, promote apoptosis, and diminish the GSIS ability of Min6 cells by suppressing the protein level of p-Akt in Min6 cells.

Our study revealed that blocking the MCP-1/CCR2 axis resulted in a partial restoration of the p-Akt protein level in pancreatic islet cells of T2DM mice, leading to a significant improvement in their proliferation and insulin secretion capacity, as well as glucose metabolism. Similar results were observed when the MCP-1 signaling was blocked in *db/db* mice [9].

### Additional pathways of glucose metabolism regulated by bone marrow adipocytes

BMAT is intricately linked to bone tissue. Within the skeletal microenvironment, adipocytes and osteoblasts originate from a common progenitor, BMSCs, and their behaviors are typically inversely related [37]. Mature osteoblasts typically restrain the adipogenesis of BMSCs through paracrine signaling. Conversely, mature BMA negatively regulate bone metabolism by inhibiting the osteogenesis of BMSCs and enhancing osteoclastogenesis [38]. In the context of T2DM, the secretion of ADIPOQ in BMA is downregulated [39], which can impede osteoblast differentiation. Meanwhile, the expression of *Pparγ* in BMA significantly increases, facilitating adipogenesis and inhibiting osteogenesis of BMSCs. In addition, we observed a downregulation of mRNA expression of *Runx2*, a crucial transcription factor for osteogenesis, in the metaphyseal bone of type 2 diabetic mice. This was accompanied by a reduction in extracellular matrix genes like *Col1a1*, *Lcn2* and *Ocn* (Fig. 1G, H). OCN and LCN2 have been shown to enhance insulin secretion, improve insulin sensitivity of target organs, and increase glucose tolerance [40–42]. Moreover, BMA can modulate glucose metabolism by influencing the body’s insulin sensitivity through adipocytokines and inflammatory factors, including LEP, RETN, and IL-6, which are secreted by BMA [43, 44]. In summary, the increase in BMAT in T2DM indirectly impairs osteoblast function, as described above, and directly influences glucose metabolism through cytokines and inflammatory factors.

Put together, in this study, we observed a substantial accumulation of BMAT in type 2 diabetic mice (Fig. 1). In addition, the mRNA expression of *Mcp-1* in BMA exhibited a remarkable increase (Fig. 3), while the secretion level of MCP-1 in the BMS showed a significant elevation (Fig. 3). Simultaneously, the mRNA expression of the MCP-1 receptor *Ccr2* in mouse islet cells displayed a distinctive rise (Fig. 3), leading to a pronounced reduction in islet cell proliferation (Fig. 2) and a notable impairment in GSIS (Fig. 2). Furthermore, we verified that the CM derived from BMAs exhibited elevated MCP-1 levels (Fig. 4). Remarkably, akin to exogenous MCP-1, this CM substantially inhibited the proliferation of Min6 cells and compromised their insulin secretion function in vitro (Fig. 4). Concurrently, we administered the MCP-1 receptor antagonist RS to diabetic mice, and this intervention partially ameliorated the phenotype of glucose metabolism disorder in the mice (Fig. 5). Through the mutual complementarity of these in vivo and in vitro experiments, we can partially elucidate the signal communication between MCP-1 originating from BMAs and islet cells. However, the most definitive approach to investigate the interaction between the two would involve the development of genetically engineered mice with targeted elimination of *Mcp-1* from BMAs. Nevertheless, due to the overlapping of gene expression profiles between BMAs and white adipocytes, the creation of such specific knockout mice poses a considerable challenge. We are hopeful that further clarity regarding the direct influence of BMAs on islet cells can be achieved through technical and methodological advancements in subsequent studies.

**Fig. 6:**
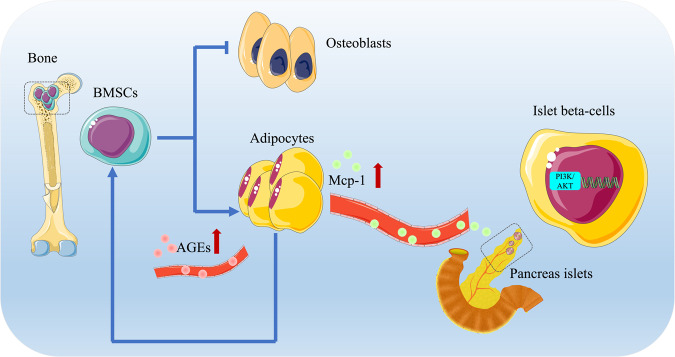
Schematic diagram of the mechanism of bone marrow adipocyte-derived MCP-1 aggravating glucose metabolism disorder in type 2 diabetes mellitus. Persistent hyperglycemia in T2DM leads to the accumulation of extracellular AGEs. These AGEs bind to their receptors, triggering a series of complex reactions that eventually lead to increased levels of BMAs and the secretion of MCP-1. MCP-1, in turn, exerts a dual impact. Firstly, it promotes the adipogenesis of BMSCs and inhibits their osteogenesis through paracrine signaling, resulting in the accumulation of BMAT. Secondly, elevated MCP-1 enters the circulation, contributing to thrombosis, inflammation, and atherosclerosis. Furthermore, MCP-1 may bind to the CCR2 receptor on pancreatic islet cells, inhibiting their proliferation and impairing insulin secretion through PI3K/Akt pathway. This complex interplay creates a detrimental “*vicious cycle*” characterized by “hyperglycemia →Mcp-1 accumulation → islet cell damage”, which promotes the progression of diabetes.

In conclusion, we assert that MCP-1 originating from BMAs is intricately linked to the onset and advancement of T2DM (Fig. 6). Nevertheless, it is essential to acknowledge that the current findings do not entirely negate the potential involvement of MCP-1 from other cell sources, which could conceivably act in synergy.

## Materials and methods

### Experimental design and animals

The experimental C57BL/6J and *db/db* mice were procured from Beijing Huafukang Biotechnology Corporation and raised in the laboratory animal center of West China Hospital. All animal experiments conducted as part of this study were approved by the Institutional Animal Care and Use Committee of the West China Hospital (Ethics Record No. 20211243A) and strictly adhered to the guide for the care and use of laboratory animals. The mice were kept in a standard 12-h light/dark cycles and provided with ad libitum access to water and corresponding diet.

Forty male C57BL/6J mice at 7 weeks of age were randomized into two groups: the T2DM group and the Ctrl group. The HFD or standard diet (SD) intervention was initiated at 8 weeks of age, following 1 week of adaptive feeding. Both HFD and SD were purchased from Beijing Huafukang Biotechnology Corporation, treated with Co60 irradiation, stored at −20 °C, and replaced every other day. The mice were continuously fed for 4 weeks. Next, the mice were fasted overnight, and the T2DM group received intraperitoneal injections of STZ (40 mg/kg, Sigma-Aldrich, USA) for 5 consecutive days, namely MLD-STZ treatment. The Ctrl group received the same amount of citrate buffer. If the ad libitum fed glucose concentration was consistently >300 mg/dL (16.7 mmol/L), the model was considered successful. Otherwise, the mouse would be excluded from the experimental group. After STZ or citrate buffer injection, the mice were fed the corresponding diet for additional 3 weeks, and IPGTT was performed as the endpoint of the experiment.

Twenty 7-week-old *db/db* mice are homozygous mutants (*Lepr*^*db*^) that spontaneously develop diabetes, which exhibited typical clinical symptoms of diabetes, such as polyphagia, polydipsia, and polyuria, as previously reported [https://www.jax.org/strain/000697]. The same number of C57BL/6J mice were used as the Ctrl group.

In the end, all mice were sacrificed to collect serum, pancreas, islet cells, eWAT, tibia, metaphyseal bone, bone marrow, and so on. Attention please, in this study, all the researchers involved in the study did not be blinded to the group of experimental animals.

### Transcriptome sequencing

The sequencing experiment process involved sequencing all mRNAs transcribed from specific eukaryotic tissues or cells during a certain period using the HiSeq platform. For library construction, the Illumina Truseq^TM^ RNA sample prep Kit method was used. Total RNA was extracted from tissues using the TRIzol (Invitrogen) method.

The operation process was carried out as follows: Firstly, magnetic beads with Oligo(dT) were used to perform A-T base pairing with ploy A to isolate mRNA from the total RNA. The mRNA was then randomly fragmented into small fragments of ~300 bp. Subsequently, the mRNA was converted into cDNA and the blunt end was made up using the End Repair Mix. An A base was then added to the 3’ end to connect the Y-shaped adapter. The products were then purified and sorted after adapter ligation, and sorted products were used to amplify by PCR, and purified to obtain the final library. The QuantiFluor® dsDNA System was used to quantify the library and mix on-board according to the data ratio. Bridge PCR amplification was performed on cBot to generate clusters, followed by Illumina sequencing.

Data analysis was performed on the Majorbio Cloud bioinformatics platform to identify and analyze the DEGs in the whole bone marrow cells from *db/db* mice, with a cut-off value of |log_2_FC| ≥ 2 (Fold change, FC) and *p* < 0.05 considered as significant difference. Gene Ontology (GO) and KEGG analyses were conducted to identify the biological functions of the DEGs and the pathways they participate in [45].

### MCP-1 receptor antagonist RS504393 intervention

After 4 weeks of HFD feeding, the mice in the T2DM group were randomly divided into two groups. One group received a subcutaneous injection of the MCP-1 receptor antagonist RS (1 mg/kg, Sigma-Aldrich) 24 h and 0.5 h before the MLD-STZ intervention, while the other group was subcutaneously injected with equivalent amount of dimethyl sulfoxide (DMSO) [46–48]. Subsequently, both groups of mice continued to undergo the MLD-STZ intervention. The Ctrl group also received a subcutaneous injection of the same amount of DMSO.

Following one week of adaptive feeding, 8-week-old male *db/db* mice were randomly divided into two groups. One group was administered RS (4 mg/kg/d), which was dissolved in drinking water at a concentration of 1% DMSO, for 8 weeks [9]. The other group received an equivalent amount of DMSO for the same duration. Mice in the Ctrl group were given the same amount of DMSO.

### Adipogenesis differentiation of bone marrow mesenchymal stem cells and OP9 cell

Mouse primary BMSCs were obtained according to the previous studies [49–53]. In Brief, the long bones of the lower limbs of mice were extracted and cut from the middle. Next, a syringe was used to rinse the bone marrow cavity thoroughly with sterile phosphate buffer solution (PBS). Then, centrifuged at 3000 rpm for 3 min. Finally, the cells were resuspended in α-Minimum Essential Medium (α-MEM, Gibco) supplemented with 10% fetal bovine serum (FBS, Gibco) and the medium was replaced every other day. The nonadherent cells were discarded and the adherent cells were passaged when they reached 80–90% confluence.

For adipogenesis differentiation, 2 × 10^5^ cells per well fourth passage cells were transferred to 6-well plates containing αMEM supplemented with 10% FBS, 1 µM dexamethasone (Sigma-Aldrich), 10 µg/mL insulin (Sigma-Aldrich), 0.5 mM 1-methyl-3-isobutanol xanthine (IBMX, Sigma-Aldrich) and 0.2 mM indomethacin (Sigma-Aldrich). After 3 days, the media was switched to α-MEM supplemented with 10% FBS, 1 µM dexamethasone, 10 µg/mL insulin and 0.2 mM indomethacin for maintenance differentiation. So repeatedly, induction ends until most cells are filled with fat droplets.

The mouse bone marrow stromal cell line OP9 was obtained from American Type Culture Collection (ATCC) and they were recently authenticated by STR profiling and tested for mycoplasma contamination. These cells were derived from the cranial cover bone of neonatal *op/op* mice and displayed typical characteristics of BMSCs [https://www.atcc.org/products/crl-2749], which were cultured in α-MEM supplemented with 20% FBS. To induce adipocytes (OP9A), the cells were treated with α-MEM supplemented with 15% knockout serum replacement (“SR”, Gibco) [54–56] when they reached 80–90% confluence.

Last, the BMSCs- or OP9-derived adipocytes were stained with oil red O (ORO; Sigma-Aldrich) to assess adipogenesis differentiation and the CM were collected.

### Isolation and purification of islet cells

The isolation and purification of islet cells were performed according to the previous studies [57–59]. Simply put, collagenase P (Roche) was dissolved in moderate precooled Hank’s balanced salt solution without magnesium and calcium (Solarbio) at a final concentration of 1 mg/mL, and then the solution was retrograde poured into the mouse common bile duct until the pancreas was fully filled. Next, the entire pancreatic tissue was quickly and bluntly cut and digested in the 37 °C water bath for 15 min. Finally, islets were purified by density gradient centrifugation and manual separation using islet specific colorant dithizone (DTZ) (Shanghai Ryon Biological Technology Corporation).

### Separation of bone marrow adipocytes and bone marrow supernatant

The methods for separating BMA and BMS were performed following a previous study [21]. Briefly, the bilateral femurs and tibias of the mice were extracted and sectioned at the midpoint of the diaphysis. The bones were positioned in a 500 μl tube with the marrow cavity aligned with the small hole at the tube’s bottom, and this tube was inserted into a 2 ml tube. The tubes were then subjected to centrifugation at 10,000 rpm at room temperature for 10 s, and this process was repeated until all the bone marrow was collected. Subsequently, 3–4 times volume of red blood cell lysate (~800 μl per mouse in total, Biosharp) was added, thoroughly mixed, and kept at room temperature for 10 minutes. Following this, the tube was centrifuged at 3000 rpm at room temperature for 3 min, and the top layer liquid (about 400 μl) was aspirated and mixed with 1 ml of PBS. The tube was centrifuged again at room temperature at 3000 rpm for 5 min, and the upper layer liquid, which is the BMA, was collected by aspirating 500 μl. The remaining 400 μl liquid in the original tube, referred to as BMS, was also collected.

### Total RNA extraction and quantitative real-time PCR

In this study, total RNA was extracted from tissues and cells using the TRIzol reagent (Invitrogen), followed by reverse transcription into cDNA using the PrimeScript^TM^ RT reagent kit with gDNA Eraser from Takara. The resulting cDNA was then amplified and quantified using TB Green Premix Ex Taq II(Takara), following the manufacturer’s instructions. The primer sequences employed in this experiment are listed in Supplementary Table 1.

The expression levels of the target genes were determined using the 2^−(ΔΔCt)^ method and normalized to the expression level of the *β-actin* gene. qRT-PCR experiments for each target gene were conducted on three independent occasions to ensure the accuracy and reproducibility of the results.

### Histopathological analysis

The tibia and pancreas of the mice were fixed in 4% formaldehyde, embedded in paraffin, and sliced into 5 μm thick slices. For the tibia, a decalcification process lasting 21 days was carried out after fixation and before embedding. Subsequently, HE staining was performed on the tibia sections. The BMAs were observed under a microscope, and changes in both number and diameter were quantified using Image J software. In addition, BrdU and TUNEL immunofluorescent staining of pancreatic islets were conducted following established protocols [59–63].

### Cell proliferation ability and apoptosis assay

Cell proliferation and apoptosis were evaluated using the cell counting kit-8 (CCK8, Dojindo laboratories, Japan) and Annexin V-FITC/PI Double Staining Apoptosis Detection Kit (Dojindo laboratories), respectively, in accordance with the manufacturer’s instructions.

### Western blot

The cells were lysed using RIPA lysis buffer, which contained a mixture of general protease and phosphatase inhibitors cocktail (Absin, Shanghai, China). The protein concentration of the lysate was measured using a BCA protein quantitation kit (Absin) and adjusted to the same concentration with 2× SDS. Equal amounts of protein samples were loaded onto 10% SDS-PAGE precast gel (ACE, Nanjing, China), and subsequently transferred to PVDF membranes (Millipore, USA). The PVDF membranes were blocked with 5% bovine serum albumin in TBS/Tween for 1 h at room temperature, and then incubated overnight at 4 °C with primary antibodies, including rabbit-anti-Akt (pan) (1:1000, Cell Signaling Technology, 4691T, USA), rabbit-anti-Phospho-Akt (ser473) (1:2000, Cell Signaling Technology, 4060T) or rabbit-anti-Beta actin (1:1000, Proteintech, 20536-1-AP, USA). The membranes were then incubated with peroxidase affinipure goat anti-rabbit secondary antibody (1:5000, Jackson ImmunoResearch, 111-035-003, USA) for 1 h at room temperature. Finally, Signals were detected using Clarity^TM^ Western ECL Substrate (Bio-Rad, USA), and the bands were visualized with the ultra-high sensitivity chemiluminescence imaging system (Bio-Rad). Beta actin was used as an internal control. In addition, the full and uncropped western blots were uploaded as “Supplemental Material”.

### Statistical analysis

Statistical analysis was conducted using SPSS 25.0, and the data were presented as mean ± standard error of the mean (SEM). The comparison between two groups was performed using an unpaired, two-tailed Student’s *t* test. Two-way ANOVA was employed for comparisons between multiple groups, and Fisher’s PLSD post-hoc test was applied when significant differences were observed. Statistical difference was taken as the cutoff value of *p* < 0.05. Statistical graphs were drawn using GraphPad Prism 8.0.

### Supplementary information

Supplementary Figure 1

Supplementary Figure 2

Supplementary Figure Legends and Supplementary Table

Supplementary material of original western blot results

## Data Availability

Raw RNA-Seq data have been deposited in the Sequence Read Archive (SRA) with accession number PRJNA1022860. All data underlying this article will be shared on reasonable request to the corresponding author.
