# Supplementary figures and images for: Pathological roles of bone marrow adipocyte-derived monocyte chemotactic protein-1 in type 2 diabetic mice
Source: Cell Death Discov. 2023 Nov 13;9:412. doi: 10.1038/s41420-023-01708-3 (PMC10643445; doi:10.1038/s41420-023-01708-3)

A

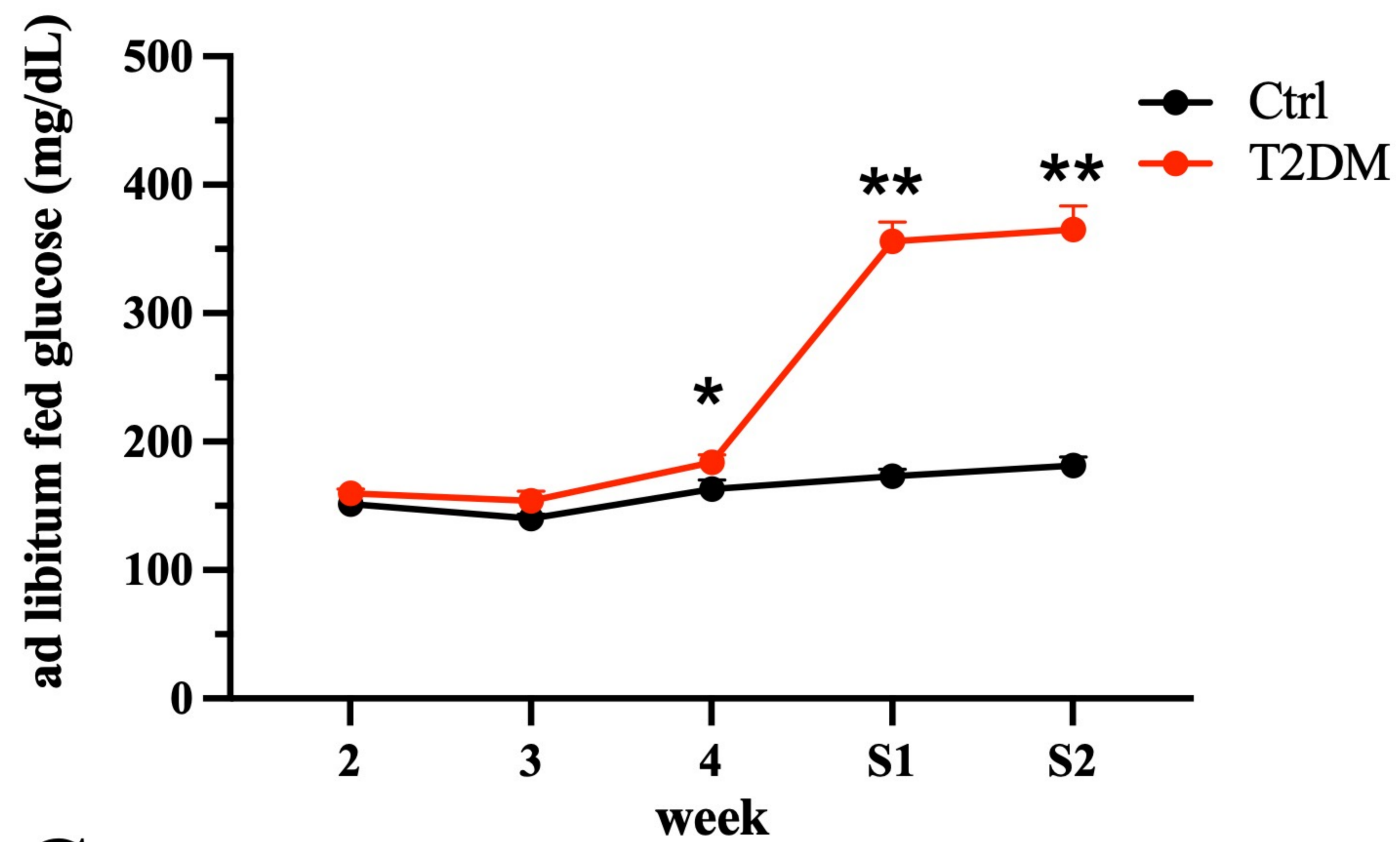

B

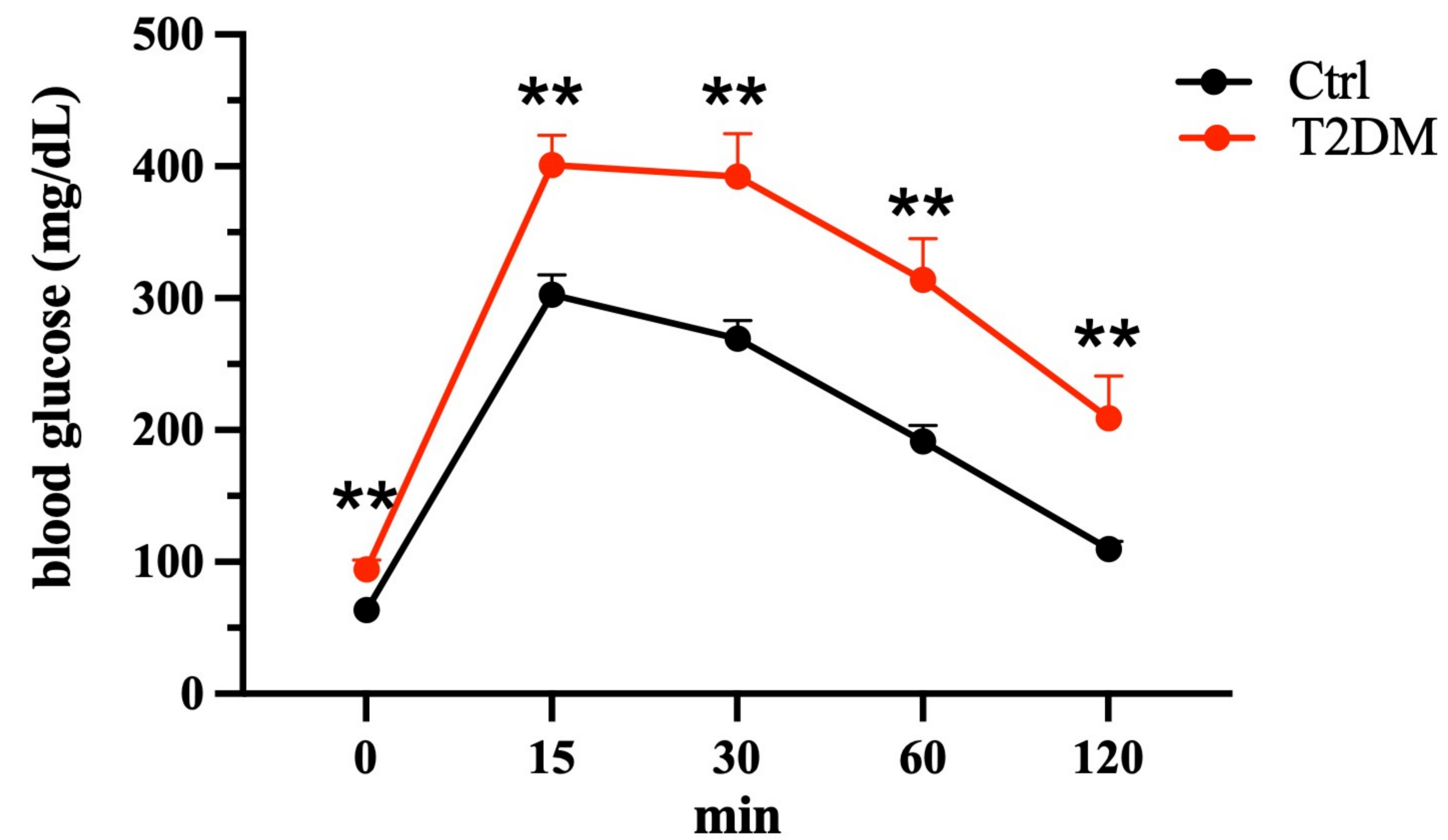

C

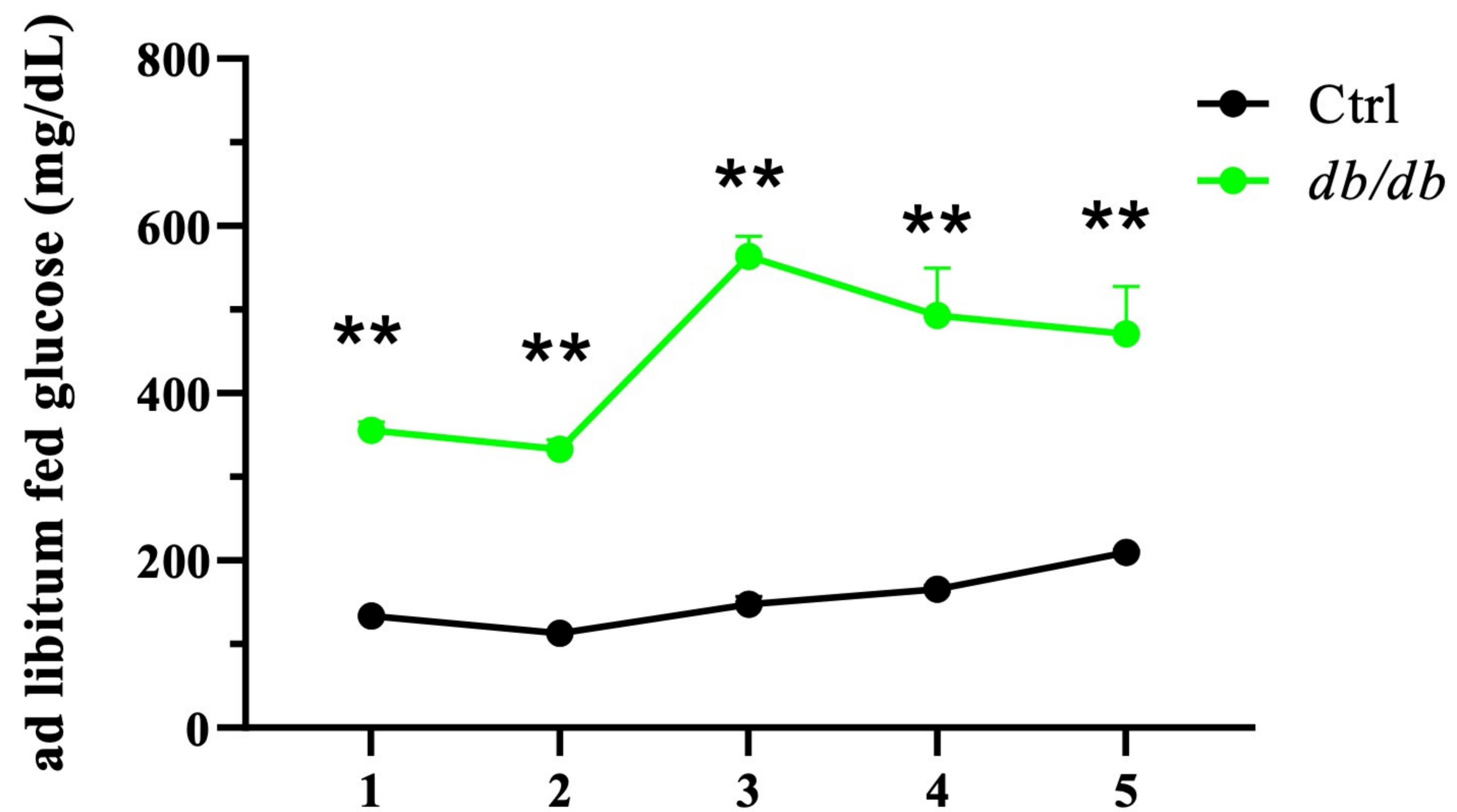

D

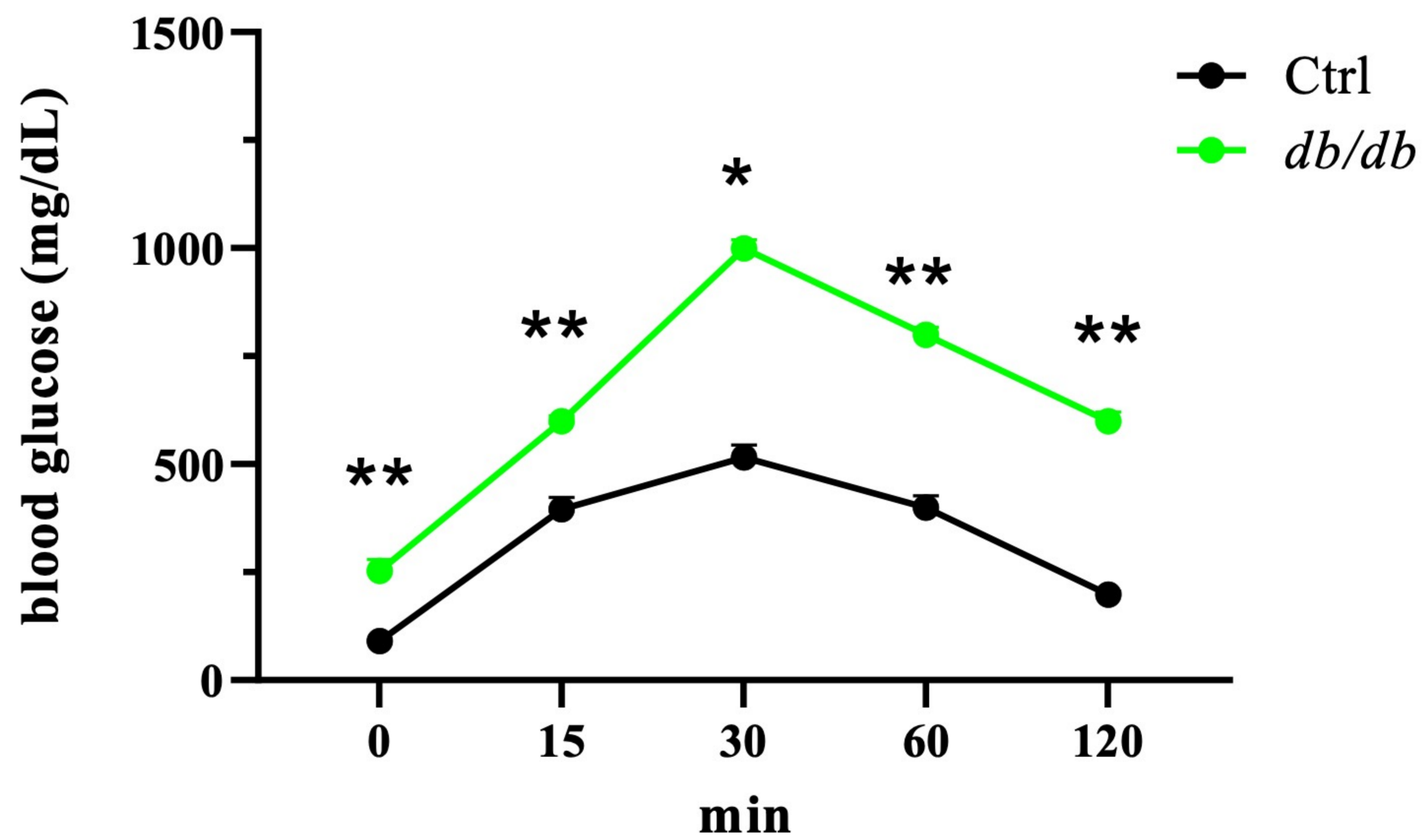

Supplement: Supplementary file 1 — Supplementary Figure 1 [file 41420_2023_1708_MOESM1_ESM.pdf]
