# Supplementary material for: Pathological roles of bone marrow adipocyte-derived monocyte chemotactic protein-1 in type 2 diabetic mice
Source: Cell Death Discov. 2023 Nov 13;9:412. doi: 10.1038/s41420-023-01708-3 (PMC10643445; doi:10.1038/s41420-023-01708-3)
Supplement: Supplementary file 3 — Supplementary Figure Legends and Supplementary Table [file 41420_2023_1708_MOESM3_ESM.docx]

**Supplementary Figure Legends**

**Supplementary Figure 1. Profound disruptions of glucose metabolism in type 2 diabetic mice**

(A) Following MLD-STZ intervention, ad libitum fed glucose levels in the T2DM mice were distinctly elevated compared to those in the Ctrl mice and this difference persisted throughout the experimental observation period (n=8). (B) Blood glucose levels in the T2DM group were markedly higher than those in the Ctrl group at each time point (n=6). (C) Ad libitum fed glucose in the *db/db* group were substantially higher than those in the Ctrl group (n=8). (D) Blood glucose levels in the *db/db* mice at each time point during the IPGTT were signally elevated compared to those in the Ctrl mice (n=6). “S1, S2, S3” refer to 1 week, 2 weeks, and 3 weeks after STZ intervention, respectively. **p*˂0.05, ***p*˂0.01.

**Supplementary Figure 2. Transcriptome sequencing of bone marrow cells from *db/db* mice**

(A) In comparison to the Ctrl group, we identified 2,915 DEGs in bone marrow cells from *db/db* mice. Among these, 1,025 genes were up-regulated, and 1,890 genes were down-regulated. (B-C) Gene enrichment analysis revealed that the primary pathways enriched in the genes differentially expressed in bone marrow cells from *db/db* group included the PI3K/Akt signaling pathway, AGE-RAGE signaling pathway in diabetic complications, chemokine signaling pathway and type Ⅱ diabetes mellitus compared to the Ctrl group. Importantly, *Mcp-1*(*Ccl2*) was involved in these pathways (n=3). (D) Further analysis of AGE-RAGE signaling pathway in diabetic complications: Persistent hyperglycemia causes non-enzymatic glycosylation of various proteins in the bone marrow, resulting in the accumulation of AGEs. These accumulated AGEs bind to their receptors, leading to the downregulation of PI3K/Akt levels and ultimately resulting in the upregulation of *Mcp-1*. This, in turn, may promote the occurrence and development of chronic complications of diabetes, including thrombosis, inflammation, and atherosclerosis. (E) Further analysis of the chemokine signaling pathway: The up-regulated chemokine in the chemokine signaling pathway includes *Mcp-1*, which binds to its receptor CCR2 and downregulates PI3K/Akt through a series of intracellular pathways. This inhibition of PI3K/Akt signaling leads to the dephosphorylation of FOXO, ultimately regulating cell proliferation, differentiation, and apoptosis. (The red circle in D and E represents up-regulation, and the blue circle represents down-regulation.)

**Supplementary Table**

**Supplementary Table 1. The primer sequences of target genes**

| **Target**  **genes** | **Forward primer (5' to 3')** | **Reverse primer (5' to 3')** |
| --- | --- | --- |
| *Adipoq*  *Pparγ*  *Tnfα*  *Il1β*  *Mcp-1*  *Insr*  *Pdx-1*  *Nkx6.1*  *Ccr2*  *Ocn*  *Lcn2*  *Runx2*  *Col1a1*  *β-actin* | ATCTGGAGGTGGGAGACCAA  CACTCGCATTCCTTTGACATC  CCCACGTCGTAGCAAACCA  GCCACCTTTTGACAGTGATGAG  CCACTCACCTGCTGCTACTCAT  AGTGCTGCTCATGCCCTAAG  ACAAATACATCTCCCGGCCC  AACACACCAGACCCACGTTC  GCCATCATAAAGGAGCCATACC  CTGTGCTGCCCTAAAGCCAA  GCCACCATACCAAGGAGCAT  AAATTAACGCCAGTCGGAGC  GGGGCAAGACAGTCATCGAA  AGATTACTGCTCTGGCTCCTAGC | GGGCTATGGGTAGTTGCAGT  CGCACTTTGGTATTCTTGGAG  ACAAGGTACAACCCATCGGC  GACAGCCCAGGTCAAAGGTT  TGGTGATCCTCTTGTAGCTCTCC  CTACTGTCCTCGGCACCATTG  GTCACCGCACAATCTTGCTC  ACTCTCCGTCATCCCCAGAG  AGGGAGTAGAGTGGAGGCAG  GACAGGGAGGATCAAGTCCCG  GGACGCCATTGGTGGTGTTA  CCACTTCTCGGTCTGACGAC  GAGGGAACCAGATTGGGGTG  ACTCATCGTACTCCTGCTTGCT |
