# Supplementary material for: Pathological roles of bone marrow adipocyte-derived monocyte chemotactic protein-1 in type 2 diabetic mice
Source: Cell Death Discov. 2023 Nov 13;9:412. doi: 10.1038/s41420-023-01708-3 (PMC10643445; doi:10.1038/s41420-023-01708-3)

Original western blots

Original western bolts of Figure 4I

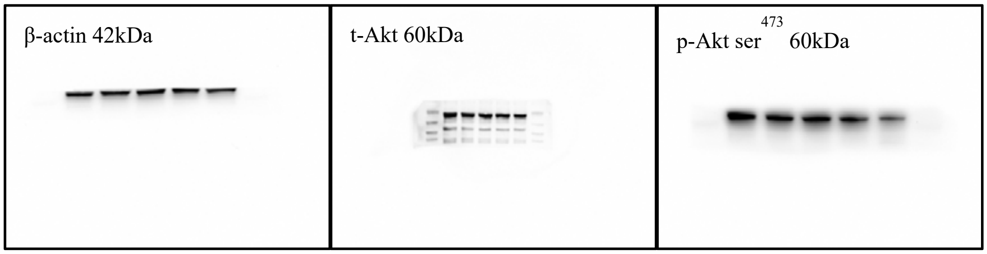

Original western bolts of Figure 4J

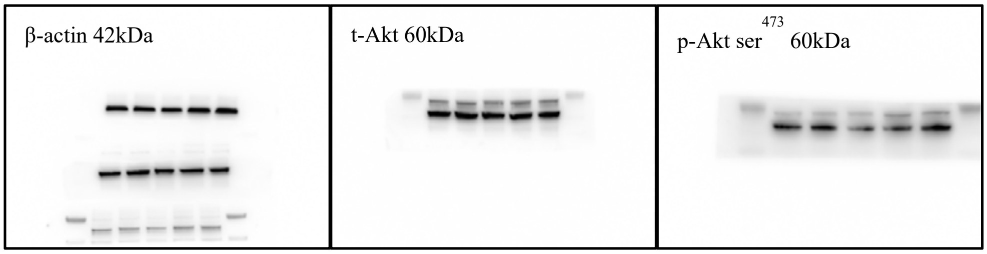

Original western bolts of Figure 4L

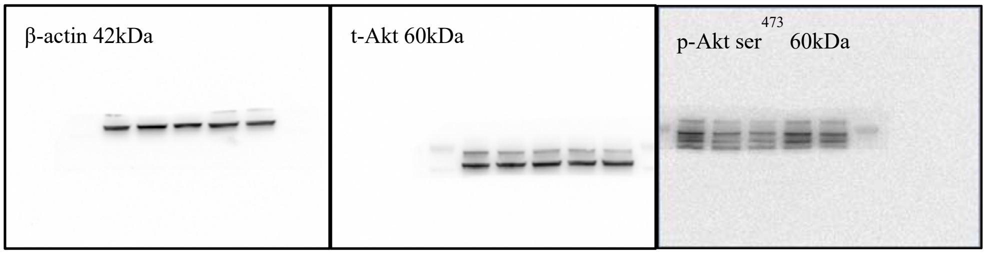

Original western bolts of Figure 5I

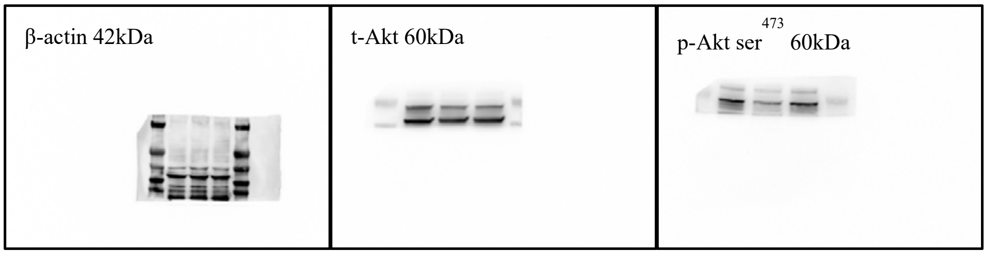

Original western bolts of Figure 5J

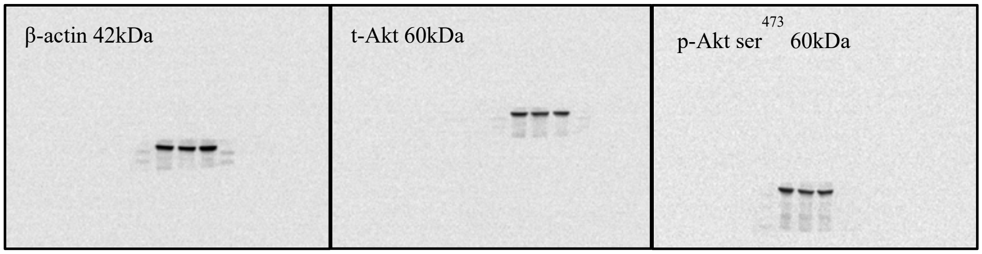

Supplement: Supplementary file 4 — Supplementary material of original western blot results [file 41420_2023_1708_MOESM4_ESM.pdf]
