# Supplementary material for: Pathological roles of bone marrow adipocyte-derived monocyte chemotactic protein-1 in type 2 diabetic mice
Source: Cell Death Discov. 2023 Nov 13;9:412. doi: 10.1038/s41420-023-01708-3 (PMC10643445; doi:10.1038/s41420-023-01708-3)
Supplement: Supplementary file 2 — Supplementary Figure 2 [file 41420_2023_1708_MOESM2_ESM.pdf]

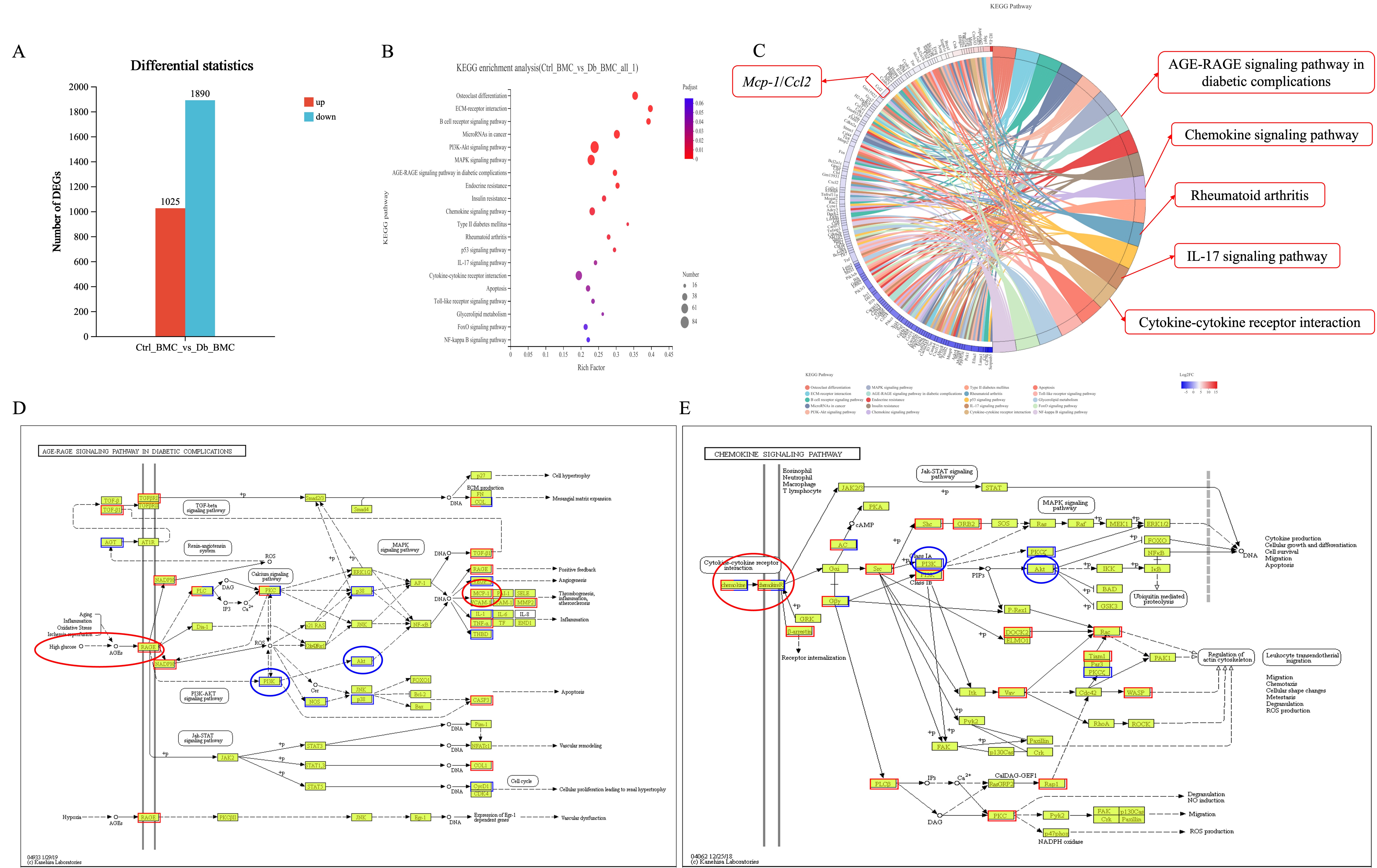

A
Differential statistics
Number of DEGs
up
down
1890
1025
Ctrl_BMC_vs_Db_BMC
B
KEGG enrichment analysis(Ctrl_BMC_vs_Db_BMC_all_1)
KEGG pathway
Rich Factor
Padjust
Number
Osteoclast differentiation
ECM-receptor interaction
B cell receptor signaling pathway
MicroRNAs in cancer
PI3K-Akt signaling pathway
MAPK signaling pathway
AGE-RAGE signaling pathway in diabetic complications
Endocrine resistance
Insulin resistance
Chemokine signaling pathway
Type II diabetes mellitus
Rheumatoid arthritis
p53 signaling pathway
IL-17 signaling pathway
Cytokine-cytokine receptor interaction
Apoptosis
Toll-like receptor signaling pathway
Glycerolipid metabolism
FoxO signaling pathway
NF-kappa B signaling pathway
KEGG Pathway
C
Mcp-1/Ccl2
AGE-RAGE signaling pathway in diabetic complications
Chemokine signaling pathway
Rheumatoid arthritis
IL-17 signaling pathway
Cytokine-cytokine receptor interaction
Log2FC
D
AGE-RAGE SIGNALING PATHWAY IN DIABETIC COMPLICATIONS
TGF-beta signaling pathway
Renin-angiotensin system
Calcium signaling pathway
MAPK signaling pathway
PI3K-AKT signaling pathway
Jak-STAT signaling pathway
Cell cycle
High glucose
AGEs
Hypoxia
Cell hypertrophy
Mesangial matrix expansion
Positive feedback
Angiogenesis
Thrombogenesis, inflammation, atherosclerosis
Inflammation
Apoptosis
Vascular remodeling
Cellular proliferation leading to renal hypertrophy
Expression of Egr-1 dependent genes
Vascular dysfunction
04933 1/29/19
(c) Kanehisa Laboratories
E
CHEMOKINE SIGNALING PATHWAY
Cytokine-cytokine receptor interaction
Jak-STAT signaling pathway
MAPK signaling pathway
Eosinophil
Neutrophil
Macrophage
T lymphocyte
Receptor internalization
Ubiquitin mediated proteolysis
Regulation of actin cytoskeleton
Leukocyte transendothelial migration
Cytokine production
Cellular growth and differentiation
Cell survival
Migration
Apoptosis
Migration
Chemotaxis
Cellular shape changes
Metestasis
Degranulation
ROS production
Degranulation
NO induction
Migration
ROS production
NADPH oxidase
04062 12/25/18
(c) Kanehisa Laboratories
